# A big data approach to improving the vehicle emission inventory in China

**DOI:** 10.1038/s41467-020-16579-w

**Published:** 2020-06-03

**Authors:** Fanyuan Deng, Zhaofeng Lv, Lijuan Qi, Xiaotong Wang, Mengshuang Shi, Huan Liu

**Affiliations:** State Key Joint Laboratory of ESPC, School of Environment, Tsinghua University, Beijing, 100084 China

**Keywords:** Environmental sciences, Energy and society

## Abstract

Estimating truck emissions accurately would benefit atmospheric research and public health protection. Here, we developed a full-sample enumeration approach TrackATruck to bridge low-frequency but full-size vehicles driving big data to high-resolution emission inventories. Based on 19 billion trajectories, we show how big the emission difference could be using different approaches: 99% variation coefficients on regional total (including 31% emissions from non-local trucks), and ± as large as 15 times on individual counties. Even if total amounts are set the same, the emissions on primary cargo routes were underestimated in the former by a multiple of 2–10 using aggregated approaches. Time allocation proxies are generated, indicating the importance of day-to-day estimation because the variation reached 26-fold. Low emission zone policy reduced emissions in the zone, but raised emissions in upwind areas in Beijing's case. Comprehensive measures should be considered, e.g. the demand-side optimization.

## Abstract (web summary)

There lacks a method to measure the rapid changes of vehicle emissions. Here the authors proposed a big data approach ‘TrackATruck’, and their estimates using the new approach show that the heavy-duty trucks (HDT) emissions of primary cargo routes/terminals were underestimated by 2–10 times in proxy-based emission inventories.

## Introduction

The accurate estimation of anthropogenic emissions is the foundation for understanding the interaction between human activities and the atmosphere. Traffic-related sources contributed 23% of CO_2_ emissions^1^ and 31% of NO_*x*_ emissions^2^ globally and up to 45% of PM_2.5_ concentrations in China’s megacities^3–5^, resulting in air pollution, global warming and ecological deterioration^6,7^. Furthermore, the variation in traffic emission is significant in both the temporal and spatial dimensions, bringing environmental effects of varying and even opposite degrees. For example, both ozone formation and loss were reported due to vehicle emissions in different environments^8–10^. Therefore, improving the quality of the traffic emission inventory requires not only accurate estimation of the total emissions but also accurate characterization of their temporal and spatial distribution.

High-quality statistical vehicle activity data and emission factors usually ensure the accuracy of the total amount, but it is more challenging to obtain a detailed distribution^11^. Taking light-duty vehicles (LDVs) as an example, by continuously improving the accuracy of emission factors and using data such as urban registered holdings and local gasoline sales to constrain the total activity level, the uncertainty regarding the total LDV emissions can be controlled to ~20%^12^. However, heavy-duty truck (HDT) trips are long-haul and frequently cross municipal boundaries, so the total amount cannot be represented by local vehicle registration or fuel sales. This uncertainty is particularly significant in countries that are rapidly advancing HDT emissions control. China, Russia, South Korea, India, Turkey and Argentina have updated HDT emissions standards two or three times in the past 10 years^13^, and so the update cycle on these standards has thus been shorter than the life cycle of HDT^14^. Therefore, the HDT fleet in these countries contains at least three emission levels (e.g., China has China III, China IV and China V). Some regions, for example, Tokyo, Saitama and Kanagawa in Japan; Mumbai, Kolkata and Chennai in India; and California in the United States, enforce stricter emissions regulations than neighbouring areas. For these regions, the fleet composition of HDT is different than that of other places. In China, Beijing implemented the China III emission standards four years earlier than other provinces. In the following years, Beijing’s China III HDT ratio for local HDT was three times higher than that in neighbouring provinces^15^. In summary, due to the rapid updating of emission standards and advanced implementation policies, China’s HDT emissions are highly heterogeneous. Thus, the total uncertainty regarding the HDT emission inventory is still large^16,17^, and the distribution caused by HDT movements continues to be difficult to evaluate. Therefore, this study focuses on this characteristic of HDTs and improves the total emissions quantity and distribution data by improving the activity data; the accuracy of emission factors is not within the scope of this study.

The key to improving the accuracy of the HDT activity dataset is converting the aggregate proxies into single descriptions of all samples. Full-sample enumeration has been used in shipping emission inventory development, e.g., the STEAM model^18,19^, but not in vehicle emission estimation. Ships frequently cross-borders, and the areas of pollutant emission are often not coincident with areas where ships are registered. Therefore, it is necessary to trace the ship emission one by one. In contrast, on-road vehicles usually run in a certain range, so the statistics based on the regional on-road vehicle population can basically reflect the emissions, especially for passenger cars. The cross-border degree of freight trucks is between ships and passenger cars. Most vehicle emission studies are based on the reported VKTs or driving behaviours of hundreds or thousands of samples^20,21^. However, if we compare the sample size to the vehicle population, the proportion of samples is usually less than 1%. Expansion from some samples to all samples introduces inestimable errors. Even though for some countries or areas, top-down inventories might use VKTs from vehicle registration data for all registered trucks in a city or province, the cross-boundary movements of trucks will introduce large errors in the actual local VKTs because the distribution of VKTs in multiple regions is unknown. In addition, although the aggregated proxies can reflect the historical regularity of vehicle activity, they are not as able to reveal the impact of irregular changes, e.g., new traffic policies. Since 2017, Beijing has successively implemented policies such as the introduction of a low emission zone (LEZ)^22^ for HDTs, the elimination of China III HDTs^23^, and the reduction in on-road bulk cargo transportation. These policies have directly limited the number of HDT activities on roads, which may have caused significant changes in HDT emissions in a short period that cannot be reflected by aggregate proxies.

This study proposes an approach called TrackATruck that has better capabilities in terms of vehicle-to-vehicle emissions evaluation. Using the TrackATruck approach with over 200 billion HDT signals from the BeiDou Navigation Satellite System (BDS)^24^, an emission inventory is established that has higher temporal and spatial resolution and lower uncertainty in the Beijing–Tianjin–Hebei (BTH) region of North China. Significant discrepancies were found between the traditional HDT emission allocation and the actual HDT emission distribution. Thus, universal time allocation parameters (proxies) of HDT emissions are refined based on the emission patterns. The effects of typical policies on HDT emissions control are evaluated. It’s found that the control of low emission zone has led to detours which caused the emissions increase in other regions.

## Results

### Emission inventory in the BTH region

**Fig. 1:**
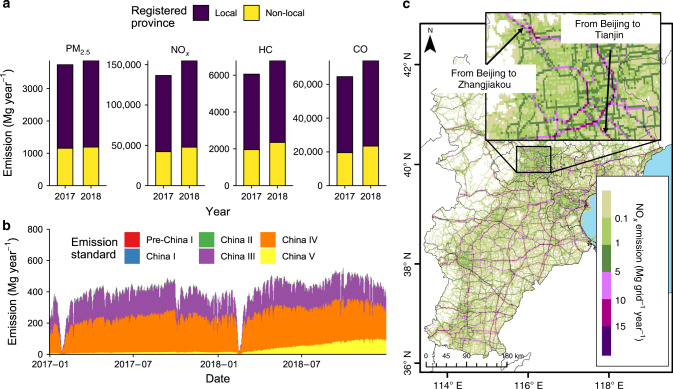
Emissions in the Beijing–Tianjin–Hebei (BTH) region by heavy-duty trucks (HDTs) in 2017 and 2018. **a** Emission amounts of four air pollutants by place of HDT registration. **b** Day-to-day NO_*x*_ emissions of HDTs and their contributions by emission standard. **c** High-resolution (0.01˚ × 0.01˚) maps of NO_*x*_ emissions by HDTs in the BTH region, 2018. The subgraph is the core area of Beijing. *Source data are provided as a Source Data file.

In this study, a TrackATruck emission model was established using low-precision, full-size vehicle travels big data to estimate fine-grain vehicle emissions (see Methods). The HDT emission inventory calculated by this method can reflect the influences of real-time driving conditions on every single HDT and the overall emissions. Based on the 200 billion BDS signals of HDTs, a high-resolution emission inventory was established for the BTH region using the TrackATruck model (Fig. 1). The BTH region, with an area of 218,000 km^2^ located in North China, has more than 100 million residents, the Chinese capital and three large ports. Therefore, this region has high demand for freight transportation. The annual PM_2.5_ emission of HDTs in the BTH region were 3739 Mg in 2017 and 3869 Mg in 2018, reflecting an increase of 3.5% (Fig. 1a). The annual NO_*x*_ emission of HDTs were 136,540 Mg in 2017 and 155,107 Mg in 2018, representing an increase of 13.6%. Approximately 31% of NO_*x*_ emissions were from non-local HDTs in 2017 and 2018. The day-to-day variation in HDT emissions in this region is very substantial (Fig. 1b). In 2018, the minimum NO_*x*_ emission of HDTs was 20.56 Mg day^−1^ on 16th Feb. (the first day of the Lunar New Year festival), and the maximum NO_*x*_ emission of HDTs was 552.31 Mg day^−1^ on 29th Sept, for a 26-fold difference. The China IV HDT is the primary subsector accounting for an average of approximately 53% of total PM_2.5_ emissions and 55% of total NO_*x*_ emissions over a two-year span. High-resolution gridded HDT emissions data in the BTH region were also presented (Fig. 1c). The HDT emissions were highly concentrated on a small number of intercity roads throughout the BTH region. The NO_*x*_ emission intensity of HDTs on these roads was generally above 5 Mg grid^−1^ year^−1^. For example, sections of the 6th Ring Road, 5th Ring Road, highways from Beijing to Tianjin, and highways from Beijing to Zhangjiakou had high emission intensities (subgraph in Fig. 1c). The HDT emissions in the western mountains of BTH were significantly lower than those in the eastern plains.

### Performance assessment of the TrackATruck model

A model validation was conducted considering two aspects. The individual vehicle result validation is introduced in the Methods section as a validation of the method itself. Here, to comprehensively evaluate the performance of the model, both the total amount and the spatial allocation were analysed or compared with those of other inventories. Recent studies with the top-down method have suggested that the PM_2.5_ emission of HDTs in BTH are in the range of 2303 to 15,200 Mg year^−1^ in 2014–2015^25,26^, while those estimated by this study is approximately 3804 Mg year^−1^ (two-year average). The difference between previous studies and this study is from −39% to 299%, with a coefficient of variation about 99%. This very large difference comes from the total VKT, fleet composition and emission factors used. Although the single-truck VKT is recorded by an odometer, it is difficult to determine how much of the driving occurred in the BTH region, which may lead to overestimation. On the other hand, omitting the emissions from non-local HDTs driving into the BTH region will result in underestimation of the emissions. Thus, the total VKT is significantly different between these studies. Here, using truck-by-truck big data, this study advances the calculation of the total VKT, the proportion of local/non-local HDT, and the fleet composition (e.g., the proportion of old trucks). Our results show that local HDTs contributed only 69% of emissions for both PM_2.5_ and NO_*x*_ over a span of two years in the region (Fig. 1a).

Several studies in Beijing used traffic-volume data to estimate the total on-road HDT emissions. Due to the uncertainty in distinguishing vehicle technology categories from traffic observations, the values calculated by different studies for HDTs can differ by three orders of magnitude (2.81 Mg year^−1^ of PM_2.5_ to 1189 Mg year^−1^ of PM_2.5_)^27,28^. In addition, some studies claimed that they used traffic-volume data to calculate high-resolution emissions inventories, but only the distribution, not the total annual HDT emissions, was provided^29,30^. The PM_2.5_ emissions of Beijing’s HDTs calculated in this study were 218.76 Mg year^−1^ (two-year average). Compared with the traffic-volume-based approach, our approach is feasible for both a large area and the city centre. In addition, the vehicle technology category (e.g., China III or IV) usually leads to a large difference in emissions, which cannot be directly identified in the traffic-volume data. This information for each individual vehicle is kept until the emission aggregation stage of the TrackATruck approach; hence, a high-resolution and detailed emission composition can be provided.

**Fig. 2:**
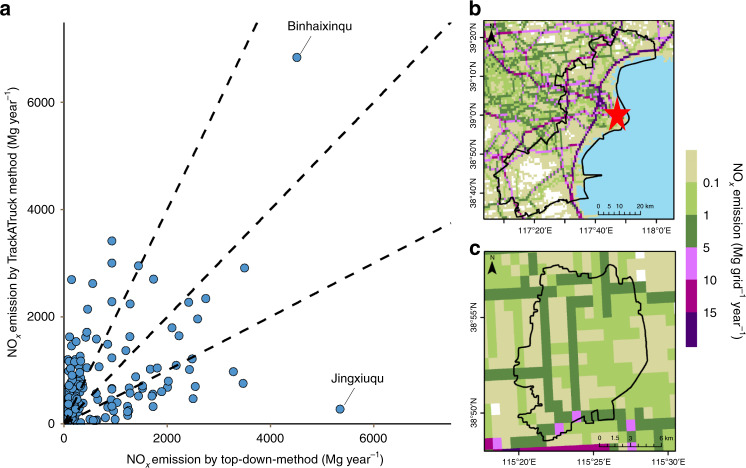
Comparison of county emission inventories obtained by the TrackATruck method with the vehicle stock obtained based on the top-down method. **a** NO_*x*_ emissions for 155 counties in the BTH region. **b** NO_*x*_ emission map (0.01˚ × 0.01˚) for Binhaixinqu obtained using the TrackATruck method. The star symbol is the location of Tianjin port. **c** NO_*x*_ emission map (0.01˚ × 0.01˚) for Jingxiuqu obtained using the TrackATruck method. *Source data are provided as a Source Data file.

The third advantage lies in the spatial distribution of emissions by TrackATruck, which is more in line with the actual characteristics of cargo activities. Figure 2a shows a comparison of the NO_*x*_ emissions of HDTs of 155 counties in the BTH region calculated by the vehicle-stock-based top-down method with those calculated by the TrackATruck method using the same emission factor. In 79% of counties, the emissions estimated by top-down method are out of the range of −50% to 150% compared to the TrackATruck results, (out of range between the dashed lines in Fig. 2), and 4% of counties which differences are more than 15 times. Further analysis was performed on the two counties (Binhaixinqu and Jingxiuqu) that have the largest differences between the two methods. It was found that based on the top-down inventory method, the emissions of Binhaixinqu and Jingxiuqu were 4512.71 Mg year^−1^ and 5305.63 Mg year^−1^, respectively. Based on the TrackATruck method, the emissions of Binhaixinqu and Jingxiuqu were 6835.57 Mg year^−1^ and 276.01 Mg year^−1^, respectively. Comparing the high-resolution emission maps of Binhaixinqu and Jingxiuqu calculated by the TrackATruck method (Fig. 2b, c), Binhaixinqu has not only a much larger area than Jingxiuqu but also multiple highways and a large port (Tianjin port). In contrast, there are few major freight corridors and no other type of transportation hub in Jingxiuqu. From the information on the map, we believe that the emissions calculated by the TrackATruck method can more reasonably reflect the distribution of HDT emissions within the BTH region.

**Fig. 3:**
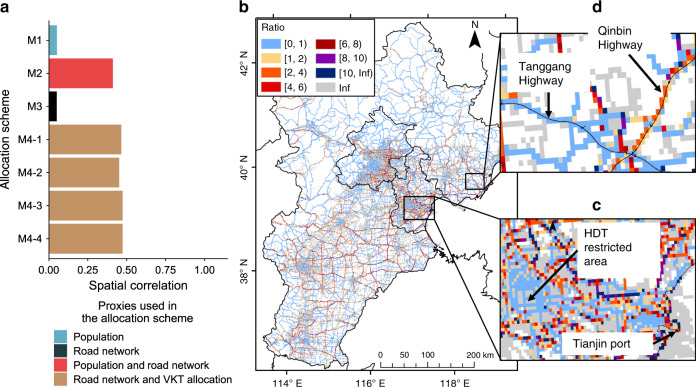
Comparison of high-resolution (0.01˚ × 0.01˚) HDT emissions calculated with the TrackATruck method and with various proxy-based allocation schemes. **a** Pearson’s correlations among NO_*x*_ emissions. M1 represents that the total emissions are allocated by population density. M2 represents that the total emissions are allocated by the road network. M3 represents that the total emissions are allocated by population density as well as the road network. M4-1 to M4-4 represent that total emissions are allocated using the road network with different VKT allocations. The total HDT emissions are set as same in all schemes. **b** The ratio of HDT emissions for each 0.01° grid between this study and the M4-1 method. **c** Tianjin Port area. **d** Two Hebei highways (Tanggang and Qinbin Highways) in b. *Source data are provided as a Source Data file.

To further identify the ability of the TrackATruck method to carry out spatial allocation, the same total emissions were allocated into 0.01° grids by seven allocation proxy schemes used in previous studies^16,17,31^, including population (M1), road density (M2), population and road density (M3) and VKT weighted road density (M4-1 to M4-4); see details in the Methods. Next, we used Pearson’s correlation to evaluate the spatial correlation between 0.01° gridded NO_*x*_ emissions of the HDTs of these schemes and the results of this study (Fig. 3a). M1 and M3 have the lowest correlation, showing that the distribution of HDT emissions has almost no correlation with the population density. The correlations of M2 (road density) and M4-1 to M4-4 (road-density-related allocation scheme with different weighting factors) to the TrackATruck results are similar but all below 0.5, indicating that none of the proxy schemes perform well. The results show that the spatial correlations are 0.47 (M4-1) and 0.45 (M4-2) and that they are slightly higher than those of M2, indicating that an appropriate VKT allocation weight can slightly improve the accuracy of the spatial allocation of HDT emissions based on the road density. M4-3 and M4-4 are the respective optimization results of M4-1 and M4-2 obtained by Ye’s method^32^ (see Methods). The spatial correlations after optimization are not significantly improved compared to those before optimization. The reasons for the low correlation between the road-density-based proxy and real-world driving were further analysed by calculating the ratio between the HDT emissions for each 0.01° grid from this study and that from the M4-1 method (Fig. 3b). One reason is that the proxy cannot reflect HDT activity with various influences in the real world. The ratio of the grids around cities and ports with more freight demand is usually greater than 1, and the ratio of some grids is greater than 10 (e.g., Tianjin port, see Fig. 3c). We further checked these grids and found that they are cargo terminals. In these grids, HDT idling or low speed travel will continue over a longer time and cause more emissions in a small area^33^. Ratios smaller than 1 appear in the regions with high road density, which are large cities such as Beijing, Tianjin and Shijiazhuang. Because these cities have a policy for long-haul truck detours, the road-density-based allocation scheme may overestimate the emissions in these regions (Fig. 3c). The other reason is that the aggregation method does not consider the variance in the busyness level for the same road type. For example, Fig. 3d shows that the two highways (Tanggang and Qinbin highways) have significantly different ratios, which indicates that due to the higher HDT traffic volume, the emissions of Qinbin highway in this study are significantly higher than that of Tanggang highway; however, in the M4-1 method, the VKT allocated values in these two highways are similar, and the emissions are not obviously different. Overall, our results show that even with the same total HDT emissions, the distribution on some primary cargo routes/terminals can be underestimated by 2–10 times in proxy-based emission inventories, while emissions on other routes are overestimated.

### Proxies for emission allocation

**Fig. 4:**
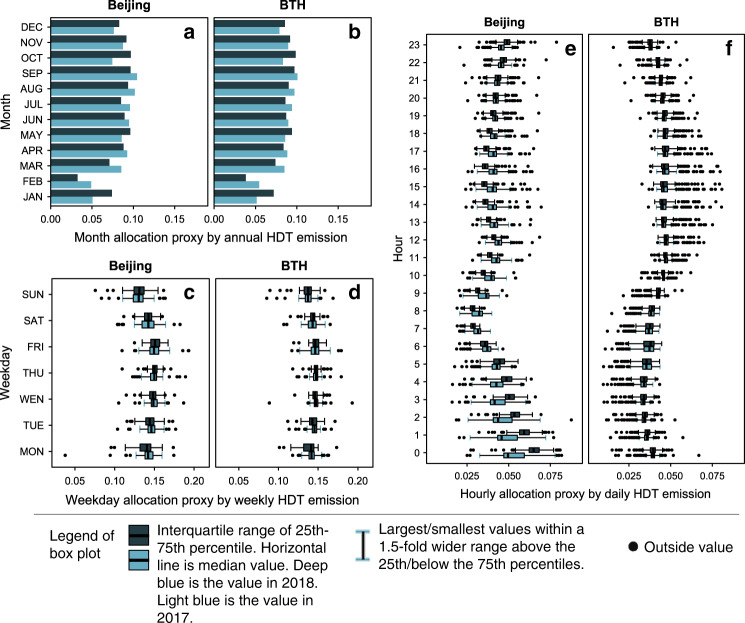
The temporally allocated proxies at various time scales obtained by using the NO_*x*_ emission data of HDTs. **a** Monthly allocation proxy in Beijing. **b** Monthly allocation proxy in the BTH region. **c** Weekday allocation proxy in Beijing. Where the *n* = 683 independent days. **d** Weekday allocation proxy in the BTH region. Where the *n* = 683 independent days. **e** Hourly allocation proxy in Beijing. Where the *n* = 16464 independent hours. **f** Hourly allocation proxy in the BTH region. Where the *n* = 16464 independent hours. Some samples during the special festivals were removed when statistics of weekday/hourly proxies were conducted, to obtain more general results. *Source data are provided as a Source Data file.

Compared to big-data analysis, the top-down approach has the advantage of being easy to apply. Therefore, we try to extract more general rules to provide parameters to improve the top-down emission inventory. For spatial allocation, our analysis above shows that no simple allocation scheme has a better correlation with big-data results. Therefore, we do not have any recommended scheme for spatial allocation. However, considering the importance of the time pattern in emission inventory studies^34^, we update the proxies of temporal allocation for Beijing and the BTH region (Fig. 4). Due to the sharp decline in HDT emissions during the Chinese Lunar New Year festival, the values of the allocated proxies in January and February are generally lower than in other months. The ratio of weekdays versus weekends is approximately 1:0.8, which is significantly smaller than the results of the California study^35^. Thursday and Friday are the busiest days, while Sunday is the lowest for freight transportation in Beijing. In Beijing, midnight is the busiest hour based on the hourly allocation, and the values during the night-time hours (23:00–6:00^+1^) of 2018 are significantly higher than those in 2017 due to the truck control policy applied during the daytime.

### Evaluation of the Beijing LEZ policy

Here, the Beijing low emission zone (LEZ) policy was evaluated using day-to-day HDT data. Beijing’s LEZ policy has been implemented since 21st Sept. 2017. In Beijing’s LEZ policy, HDTs are divided into three categories: completely banned HDTs, which cannot enter the LEZ (the area within the 6th Ring Road in Beijing) at all throughout the day, including pre-China I, China I/II HDTs and China III non-local HDTs; partially banned HDTs, which can enter the LEZ only during the period from 23:00 to 06:00^+1^, including China III local HDTs and China IV/V non-local HDTs; and non-banned HDTs, which are free to enter the LEZ, including only Beijing’s locally registered HDTs, which can meet the China IV/V standards.

**Fig. 5:**
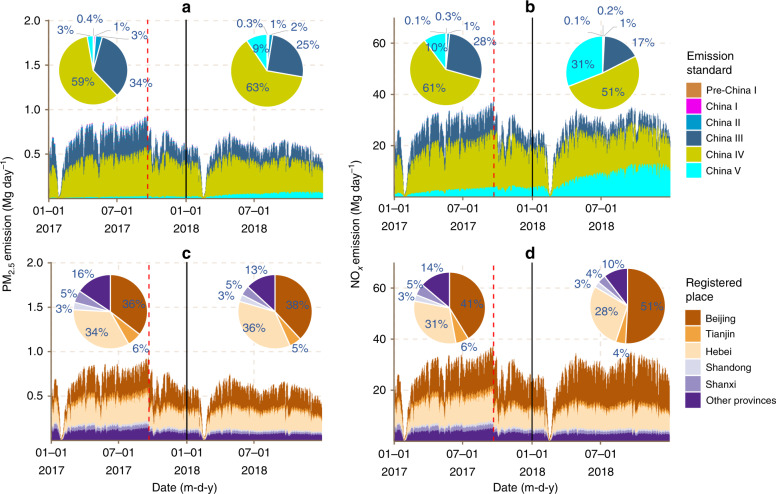
Day-to-day PM_2.5_ and NO_*x*_ emissions of HDTs in Beijing (area chart) and their contributions by various statistical patterns (area and pie chart). **a** PM_2.5_ emissions by emission standard. **b** NO_*x*_ emissions by emission standard. **c** PM_2.5_ emissions by registered place. **d** NO_*x*_ emissions by registered place. The pies show the contributions of the annual emissions in several different places and emission standards. The red dashed lines indicate the date of LEZ implementation. *Source data are provided as a Source Data file.

According to the policy requirements, most of the China III HDTs are banned. Therefore, the China III HDT emissions in Beijing dropped rapidly after the implementation of the LEZ (Fig. 5a, b). Similarly, all non-local HDTs are subject to LEZ controls; thus, the emissions of non-local HDTs have also been rapidly reduced after the implementation of the LEZ, and the daily emissions thereafter are lower than the levels before the implementation of the policy. This study also analysed the time series of HDT emissions of another city near Beijing but found no similar emissions changes during the same period (e.g., Fig. 1b). Therefore, we believe that the changes in HDT emissions are unique to Beijing and are likely to be attributed to Beijing’s LEZ.

We compared HDT emissions in whole Beijing in the same months before (1st Jan. to 20th Sept. 2017) and after (1st Jan. to 20th Sept. 2018) the implementation of LEZ. The NO_*x*_ emissions in the period are 6996.11 Mg in 2017 and 6729.83 Mg in 2018, and the PM_2.5_ emissions in the period are 179.09 Mg in 2017 and 142.09 Mg in 2018. The smaller NO_*x*_ emission changes (4% of decreases) are mainly attributed that the LEZ indirectly led to the increase in emissions of China V local HDTs. The NO_*x*_ emissions of China V HDTs increased from 959.48 Mg in 2017 to 2980.02 Mg in 2018, an increase of 3.11 times. The PM_2.5_ emissions of China V HDTs increased from 6.81 Mg in 2017 to 18.40 Mg in 2018, an increase of 2.7 times. The local China V HDTs are the main contributors to the increased emissions in all China V HDTs, with a share of approximately 85% for both pollutants (NO_*x*_ and PM_2.5_). The statistical data show that the number of newly registered HDTs in Beijing in 2018 was 2.7 times higher than the average of the past five years. In contrast, the numbers of new HDTs in Tianjin and Hebei in 2018 were similar to those of the past 5 years. This result occurs because Beijing implemented the China V emission standard for HDTs before 2017^23^, while Tianjin and Hebei did not. Thus, by purchasing new HDTs in Beijing, a freight company can have more non-banned HDTs. In fact, the transportation demand did not decrease with the increase in truck control. Beijing’s GDP and road freight volume did not change significantly from 2017 to 2018. According to the data from this study, the total VKT of all HDTs in Beijing in 2017 was 3338.79 million km, and in 2018, it was 3594.43 million km, a gap of only 7%. Thus, it can be considered that the demand for HDT use in Beijing did not change significantly in those 2 years. The LEZ prevents a certain group of HDTs from entering urban areas, and the most likely result is that non-banned HDTs fulfil the demand formerly provided by the banned HDTs. Overall, the policy resulted in lower PM_2.5_ emissions in Beijing but not lower NO_*x*_ emissions. The NO_*x*_ emission factors highly rely on the correct usage of a selective catalyst reduction (SCR) system. Without further reports on real-world SCR use cases, we did not reduce the NO_*x*_ emission factor for China V HDTs from China IV^36^.

**Fig. 6:**
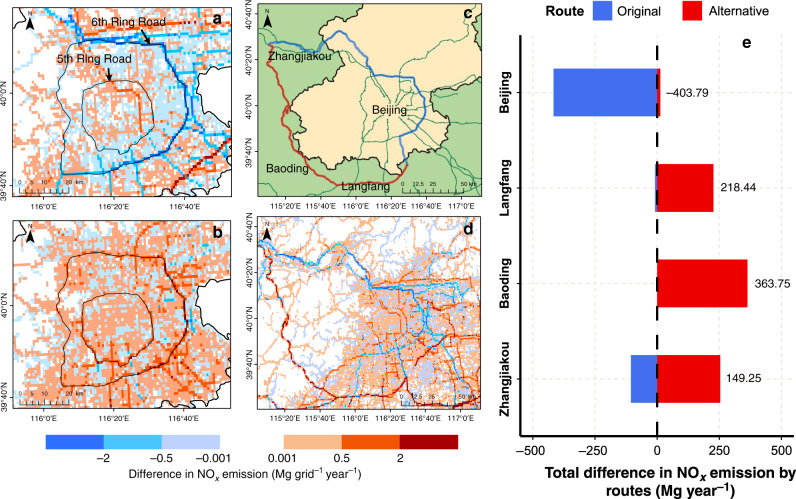
Differences in the high-resolution (0.01˚ × 0.01˚) maps of NO_*x*_ emissions by HDTs between 2017 and 2018. **a** Two-year differences in emissions by non-local HDTs in the core of Beijing. The two black lines circling the city are the 5th Ring Road and 6th Ring Road (LEZ’s range), which are identified by arrows and labelled. **b** Two-year differences in emissions by local HDTs in the core of Beijing. **c** Illustration of the alternative route (red line) and original route (blue line) in the LEZ. The names and locations of the four cities along these two routes are labelled in black. **d** Difference in gridded NO_*x*_ emissions of HDTs along the alternative and original routes over two years. **e** Comparison of the total differences in NO_*x*_ emissions by HDTs between the four cities in two years. The numbers in (**e**) represent the total net differences in HDT emissions. *Source data are provided as a Source Data file.

More specifically, LEZ aims to control HDT emissions inside the Beijing’s 6th Ring Road (Fig. 6a). Thus, the NO_*x*_ emissions inside the 6th Ring Road from non-local HDTs decreased from 2128.58 Mg year^−1^ in 2017 to 1628.11 Mg year^−1^ in 2018, a decrease of 500.47 Mg. However, the local HDT emissions increased to fill the capacity gaps left by the non-local HDTs (Fig. 6b). The NO_*x*_ emissions inside the 6th Ring Road from local HDTs increased from 2288.40 Mg year^−1^ in 2017 to 2768.77 Mg year^−1^ in 2018, an increase of 480.37 Mg. In summary, HDT NO_*x*_ emissions inside the 6th Ring Road (including the 6th Ring Road itself) are 4416.98 Mg year^−1^ in 2017 and 4396.88 Mg year^−1^ in 2018, the decrease of only 0.5%. PM_2.5_ emissions inside the 6th Ring Road (including the 6th Ring Road itself) are 110.72 Mg year^−1^ in 2017 and 86.39 Mg year^−1^ in 2018, which decreased by 22%. According to the change of local/non-local HDT emissions, up to 96% emissions reduction benefits of non-local HDTs are offset by the increase in the emissions of local HDTs.

For the intercity HDT emissions, the change in the spatial distribution is due to HDTs detouring around the city due to the LEZ (Fig. 6c). The original route goes through the 6th Ring Road of Beijing, which is short and usually uncongested. However, due to the LEZ restrictions, HDT drivers chose to detour through the three cities west and south of Beijing, causing the NO_*x*_ emissions along the corresponding routes to increase significantly between the two years (Fig. 6d). The net changes in the alternative route and the original route were 857.72 Mg and −530.07 Mg, respectively. Overall, 79% of the reduction in HDT emissions from the original route occurred in Beijing, while an increase in HDT emissions on the alternative route occurred in the other three cities (Fig. 6e). The alternative routes with increased HDT emissions were located southwest of Beijing. Historical observations show that the more severe haze in Beijing mostly occurs under meteorological conditions dominated by a southwest wind^37–39^. Therefore, although the LEZ shifted the HDT emissions far from urban areas, the detours might lead to more NO_*x*_ emissions occurring in an adverse position and impacting Beijing’s air quality standards. Thus, the final air pollution impact of LEZ on Beijing’s primary air pollutant dispersion and secondary pollutant formation is complicated and influenced by more than inner-city HDT emissions.

## Discussion

The current top-down emission inventories are mainly driven by statistical data, which creates challenges in improving the resolution, and input data are usually delayed by several months. Here, we develop a big-data-driven approach and demonstrate both the capability of the method and the advantages of the high-resolution truck trajectory big data. We estimate the day-to-day HDT emission in the BTH region for 2017 and 2018; our results suggest the importance of including non-local HDT emissions in local emission inventories. Both the total emissions amount and their distribution are better estimated with higher resolution under this approach. Temporal profiles for monthly, daily and hourly emissions allocation were summarized for application in a top-down emission inventory. However, for spatial allocation, our analysis indicates that no simple allocation scheme has good correlation with the big-data results. The TrackATruck system can tolerate different BDS or GPS data sampling frequencies using the similarity calculation module.

This study offers novel insights into local policy evaluation and suggests the need for regional joint control strategies. In Beijing, the LEZ promotes the updating of HDT fleets within the restricted area, but the demand for HDTs has not changed. Thus, approximately 96% of the emission reduction benefit from restricted HDTs was offset by an increase in unrestricted vehicles in the restricted area. From a regional perspective, it is difficult to achieve a win–win result on emission control through this restriction for local and surrounding areas, as the long-haul HDT detour caused by the LEZ may increase HDT emissions in neighbouring cities. In Beijing, those neighbouring cities are the upwind regions when severe air pollution occurs. Thus, when considering the long-range transport of air pollutants, the overall air quality benefit is still in doubt. In addition, this seesaw effect creates a new problem of responsibility allocation for HDT emissions control among multiple cities.

Environmental research aimed at protecting public health needs to reflect the high concentration of pollutants in hot spots rather than just the long-term and large-scale average. Schools, hospitals, transportation hubs and so on are more vulnerable to dynamic traffic emissions, but traditional environmental statistics are clearly not enough to protect the population in these areas. Moreover, the coverage density of traffic and environmental monitoring networks is not sufficient to indicate the impact of traffic emissions on each sensitive area. Therefore, it is necessary to introduce traffic big data to build a fine-grained picture of dynamic emissions. However, no large-scale system collects vehicle position data every second, although it is the most suitable data source for emission simulation, so there is a clear need for a bridge method worldwide. Our study establishes a bridge from low-precision but full-size vehicle travel big data to fine emissions. TrackATruck can be promoted in countries and regions where information-related technology and vehicle monitoring systems have been developed. For example, large-scale implementation of vehicle navigation systems run by third parties or manufacturers and other monitoring platforms could be prerequisites for enabling application of this model to a region. These prerequisites exist in many countries, including the United States, Europe, Japan, South Korea, etc. Maintaining personal privacy in this type of data collection is a major cause of public concern, but this problem is not insurmountable, as the current technology is able to erase personal information in the process of collecting data or providing data to researchers. The key is whether the third-party platforms, vehicle manufacturers, or freight companies that own the data are willing to do such additional work, as it is not profitable. However, if incentives or lobbying strategies promoting environmental public welfare can be used to motivate companies, it is possible to obtain vehicle travel big data with personal information erased. This is exactly the process in which this research is realized. If these big data can be used and combined with the TrackATruck method, it can provide more dynamic vehicle emissions data.

Our study also demonstrates how large the difference can be in vehicle emissions estimation when using different approaches. The difference of regional HDT emissions is significant between the several studies. For county-level emissions, the difference in individual counties by different methods can reach more than 15 times. Even if total amounts were set the same, the top-down methods also underestimated HDT emission on primary cargo routes/terminals by a multiple of 2–10 and overestimated those on other routes. The day-to-day emissions revealed that certain special events, including new control policies and holiday effects, may cause dramatic changes in HDT emissions. Even for regions that cannot implement the same method for various reasons, this result is still useful as it can help them estimate the uncertainty of air pollution sources. In addition, more than 200 cities globally have implemented vehicle traffic control to varying degrees^22^. The analysis of Beijing’s LEZ in this study can be a reference to formulate more comprehensive vehicle emission control measures in these places.

This study has some uncertainties because of the imperfect input data. First, the BDS big data do not cover 100% of all HDTs at all times. Based on our QA/QC analysis (see Methods), there is still a 30% chance of HDT data loss due to vehicles not being equipped with the BDS or incorrect data transfer. Even for those trucks in the database, shielded BDS signals in mountainous areas and specific fields can lead to the underestimation of HDT emissions in these areas. In addition, the emission factors, which were not improved in this study, still have some uncertainty.

## Methods

### Data description and QA/QC

The scope of this study is HDT emissions in the BTH region of China. The population distribution data are from the LandScan database. The HDTs defined in this study refer to trucks with a gross weight of more than 12 tons. The GDP, freight volume and new HDT registration of Beijing are from the database of the National Bureau of Statistics of China (NBSC). The source of the BDS data for this study is the Nationwide Road Freight Vehicles Public Supervision and Service platform of SINOIOV (SINOIOV platform). HDT BDS data in the BTH region on the SINOIOV platform were collected, and the time range was 1st Jan. in 2017 to 31st Dec. in 2018. The main function of the SINOIOV platform is to support the digital management of road freight transportation in China. Currently, nearly 6 million trucks have been connected to the SINOIOV platform, covering 96% of medium/HDTs used in the Chinese freight market. However, some trucks did not report data for various reasons, e.g., policy controls, old truck retirement and not reported to the government. Therefore, this study further checked the coverage of HDTs by performing a QA/QC analysis on the raw data. We counted the number of HDTs collected from the SINOIOV platform and compared this number with the HDT registration data in NBSC. The number of HDTs from SINOIOV versus that from the government database is approximately 0.7:1, the difference stemming from the features of these two databases. For the SINOIOV database, only active trucks were registered and could report data. For the government database, both active and rarely used trucks were included. Therefore, we believe 70% is the real ratio of HDTs registered in the government databases that are still active. More than 80% of the trajectories had BDS sampling frequencies higher than 1/30 Hz. Less than 0.01% of HDTs had missing information in terms of the registration place and year, making it impossible to judge their emissions category. Therefore, the related trajectories of these trucks were excluded from the calculation, but they account for less than 0.01% of the total trajectory number.

### TrackATruck approach for HDT emissions

**Fig. 7:**
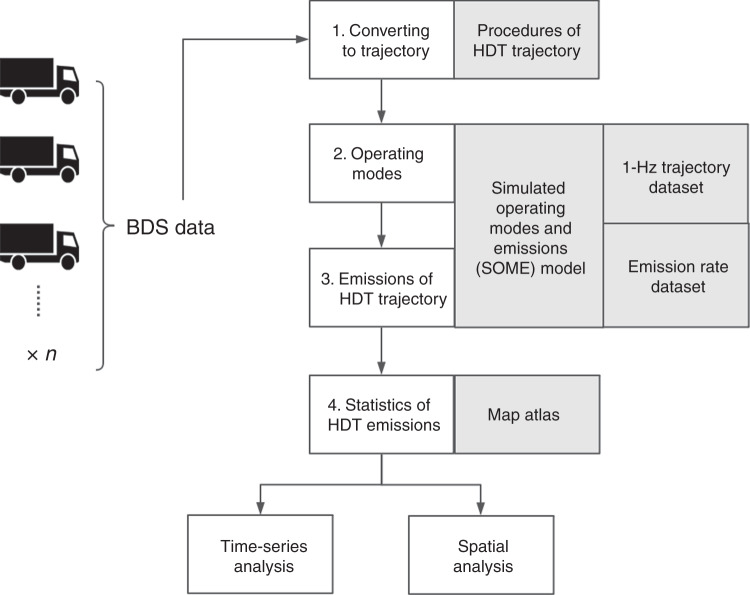
The TrackATruck framework for the HDT emission calculation used in this study. Step 1 is to convert HDT BDS data to the trajectories. One trajectory contains multiple continuous BDS data. Step 2 is to calculate the distribution of the operating modes for each trajectory, and Step 3 is to calculate the emissions for each trajectory. The simulated operating modes and emissions model is used to simulate the distribution of the operating modes by speed distribution of a trajectory and calculate emissions based on the emission rates of the operating modes. Step 4 is to aggregate that HDT emissions by all HDT trajectories, combining with various maps, e.g. provincial border, to the temporal-spatial characteristics.

TrackATruck is a bottom-up approach that considers the individual differences in trucks and constructs a high-resolution emission inventory. Previous studies have shown that the instantaneous emission rate is greatly influenced by the instantaneous driving state, such as the driving speed, acceleration, and vehicle specific power (VSP). Based on the driving speed, acceleration, and VSP, the US EPA MOVES model^40^ divides the stable driving states of a vehicle into 23 operating modes (named Opmodes in the following text). The Opmodes in TrackATruck were defined in the same way as in the US EPA MOVES model. Thus, TrackATruck must build a link from low-frequency BDS data (1/30 Hz) to the estimated Opmodes, which are commonly calculated by 1-Hz GPS data^41,42^. The framework used in TrackATruck is shown in Fig. 7.

In the first step, several continuous BDS signals of HDTs were treated as a trajectory of 300–400 s. According to our analysis of the BDS sampling frequency, the trajectory contained approximately 10 BDS signals with a sampling frequency of 1/30 Hz. Each trajectory from the BDS signals of an HDT (referred to as the 1/30-Hz trajectory in the following text) must start from the last BDS record of the previous trajectory.

In the second step, the model estimates the Opmodes distribution of the 1/30-trajectory based on the similarity between the 1/30-Hz trajectory and 1-Hz HDT GPS trajectory (called the 1-Hz trajectory in the following text) dataset in the simulated Opmodes and emissions (SOME) model. The SOME model has a database with 1-Hz GPS data, which were derived from the database given in our previous research^43–46^. In this database, multiple 1-Hz trajectories were selected based on the speed distribution of the target trajectories of the 1/30-Hz trajectory. The condition for selection is that the mean speed of 1-Hz trajectory full-in the upper and lower limits of the 95% confidence interval of the speed distribution of the 1/30-Hz trajectory. Then, the SOME model uses the following equation to calculate the distribution of Opmodes for a 1/30-Hz trajectory based on the selected 1-Hz trajectories:

1 $$\left[ {\begin{array}{*{20}{c}} {F_{m,1}} \\ \vdots \\ {F_{m,23}} \end{array}} \right] = \frac{1}{n}\left[ {\begin{array}{*{20}{c}} {f_{1,1}} & \cdots & {f_{1,n}} \\ \vdots & \ddots & \vdots \\ {f_{23,1}} & \cdots & {f_{23,n}} \end{array}} \right]\left[ {\begin{array}{*{20}{c}} {S_{m,1}} \\ \vdots \\ {S_{m,n}} \end{array}} \right]$$

where *m* is a 1/30-Hz trajectory. *F*_*m*,1_ to *F*_*m*,23_ are the simulated frequencies of 23 Opmodes of *m*. The matrix of *f*_1_ to *f*_23_ includes the Opmode frequencies of the matched n 1-Hz trajectories which. *S*_*m*,1_ to *S*_*m*,*n*_ are the similarity weights of the fitted n 1-Hz trajectories for the 1/30-Hz trajectory *m*. The following logic equation was used to calculate the similarity weight:

2 $$S_{m,n} = \left\{ {\begin{array}{ll} \frac{{\nu _{{\rm{max}}} - \mu _{{\rm{min}}}}}{{\mu _{{\rm{max}}} - \mu _{{\rm{min}}}}} & \quad\quad\quad {\rm{if}}\;\mu _{{\rm{min}}} \in \left[ {\nu _{{\rm{min}}},{{\infty }}} \right] \\ \\ \frac{{\mu _{{\rm{max}}} - \nu _{{\rm{min}}}}}{{\mu _{{\rm{max}}} - \mu _{{\rm{min}}}}} & \quad\quad\quad\quad {\rm{if}}\;\mu _{{\rm{max}}} \in \left[ { - {{\infty }},\nu _{{\rm{max}}}} \right] \\ \\ \frac{{\nu _{{\rm{max}}} - \nu _{{\rm{min}}}}}{{\mu _{{\rm{max}}} - \mu _{{\rm{min}}}}} & {\rm{if}}\;R_\nu \subseteq R_\mu \\ \\ \frac{{\mu _{{\rm{max}}} - \mu _{{\rm{min}}}}}{{\nu _{{\rm{max}}} - \nu _{{\rm{min}}}}} & {\rm{if}}\;R_\mu \subseteq R_\nu \\ \\ 0 & {\rm{if}}\;R_\nu \not\subseteq R_\mu \end{array}} \right.$$

where *μ*_max_ and *μ*_min_ are the upper and lower limits of the 95% confidence interval of the speed distribution of the 1/30-Hz trajectory *m*.*ν*_max_ and *ν*_min_ are the upper and lower limits of the 95% confidence interval of the speed distribution of the 1-Hz trajectory *n*. These limits were calculated by the mean and standard deviation of the speed along the trajectory. *R*_*μ*_ is the set of *μ*_max_ and *μ*_min_. *R*_*ν*_ is the set of *ν*_max_ and *ν*_min_. The unit for all speed values is m s^−1^.

In the third step, the model calculates the emission rates of each trajectory based on the distribution of the Opmodes and the technical parameters of the HDT.

3 $$E_{m,j} = t_m \times \mathop {\sum}\limits_{i = 1}^{23} {\left( {{\mathrm{er}}_{i,j} \times {\mathrm{BIN}}_{m,i}} \right)}$$

4 $${\mathrm{BIN}}_{m,i} = \frac{{F_{m,i}}}{{\mathop {\sum}_{i = 1}^{23} {F_{m,i}} }}$$

where *E*_*m*,*j*_ is the emissions of the 1/30-Hz trajectory *m* and pollutant *j*. *t*_*m*_ is the duration of the 1/30-Hz trajectory *m*, the unit is s. er_*i*,*j*_ is the emission rate of Opmode *i* and pollutant *j*, the unit is g s^−1^. BIN_*m*,*j*_ is the normalized frequency of the simulated Opmode.

The emission rates of Opmodes for all HDT categories are estimated based on the HDT emission factors from the emission inventory (EI) guidebook by the Ministry of Ecology and Environment of the People’s Republic of China^47^ and the portable emission measurement system (PEMS) data of the on-road truck examined in our previous works^45,46^. The EI guidebook provides the recommended HDT emission factors for guiding HDT emission inventories in China (Table 1). To map the HDT emission factors to the emission rates of MOVES’s Opmodes, we established the Opmode emission rates for a benchmark HDT model based on our PEMS data. Next, we estimated the Opmode emission rates of other HDT models based on the relationship of emission factors between the benchmark and other HDT models (Table 1).

**Table 1:** Emission factors of all HDT categories.

Fuel	Emission standard	Emission factor (g km^−1^)			
		CO	HC	NO_*x*_	PM_2.5_
Diesel	Pre-China I	13.60	4.08	13.82	1.32
	China I	5.79	0.90	9.59	0.62
	China II	3.08	0.52	7.93	0.50
	China III	2.79	0.25	7.93	0.24
	China IV	2.20	0.13	5.55	0.14
	China V	2.20	0.13	4.72	0.03
Gasoline	Pre-China I	123.13	6.75	5.81	0.29
	China I	75.79	6.76	2.98	0.16
	China II	23.32	3.00	2.90	0.07
	China III	10.71	1.35	1.71	0.04
	China IV	4.50	0.55	0.91	0.04
	China V	4.50	0.55	0.68	0.04
Others	Pre-China I	18.70	3.84	21.16	0.29
	China I	15.14	3.20	16.80	0.16
	China II	12.11	2.86	13.06	0.07
	China III	6.36	1.72	9.32	0.04
	China IV	4.67	1.19	6.52	0.04
	China V	4.57	1.19	3.73	0.04

The fourth step uses statistics for the HDT emissions based on the emissions of the HDT trajectories and map information to generate time series and maps of HDT emissions. For each HDT trajectory, we allocate the emissions of a trajectory to grids by the time duration.

### Model validation

**Fig. 8:**
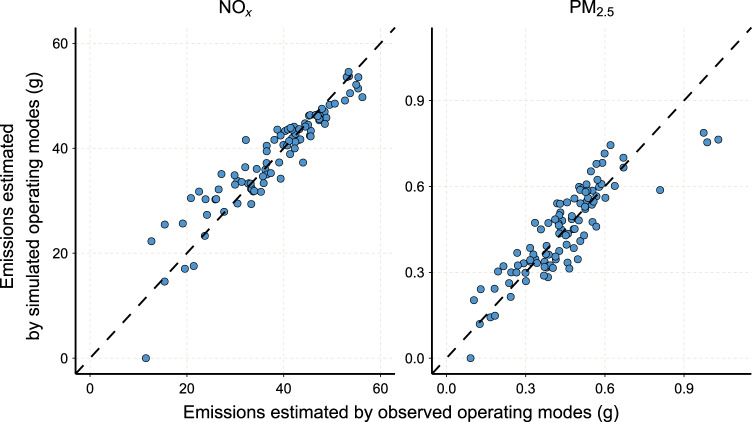
The correlations of the estimated NO_*x*_ and PM_2.5_ emissions of HDT trajectories between the observed and simulated operating modes. Each point represents that a HDT trajectory. The slops of the dashed lines are 1. *Source data are provided as a Source Data file.

This study validates the second and third steps in the TrackATruck model with real-world measurements from five HDTs. We use the SOME model to calculate the simulated Opmode distribution of the test trajectories and then calculate the pollutant emissions of the trajectory by the actual and simulated Opmode distributions, respectively. Figure 8 shows the estimated emission distributions in the test data. For NO_*x*_ and PM_2.5_, the calculated emissions of the two Opmodes are better correlated, and Pearson’s correlations are 0.93 and 0.87, respectively. The slopes of the linear fits of the two results are 1.04 (NO_*x*_) and 0.95 (PM_2.5_), indicating that the average ratio of the two results is close to 1:1. The mean absolute percentage error (MAPE) of the two results is 9.47% (NO_*x*_) and 16.75% (PM_2.5_), indicating that the relative differences between the two results are nonsignificant. Overall, there are small differences between the simulated Opmodes and the actual Opmodes when calculating the emissions of an HDT trajectory, which indicates that the SOME model can better estimate the emissions under the Opmode-based model based on the speed distribution of the HDT trajectory.

### Test of different aggregated proxies for spatial allocation

To compare our high-resolution approach with top-down methods, this study allocates the same total emissions into 0.01˚ grids by different proxy schemes. The proxy descriptions for these aggregation methods are shown in Table 2. First, for each scheme, we calculated the proxy’s value for each grid, e.g., the population in a 0.01° grid. Second, for each scheme, we converted these values into the allocation weights for each grid based on the total proxy value in the BTH region. Third, we allocated the total HDT emissions to 0.01˚ grids based on the allocation weights for each scheme. Finally, we calculated Pearson’s correlation of the 0.01˚ HDT emissions between the aggregated proxy methods and the BDS big-data method of this study. The proxy descriptions for these aggregation methods are shown in Table 2.

**Table 2:** Descriptions of the seven HDT emissions allocation methods.

Method	Proxy scheme
M1	Density of population
M2	Density of road network (road length for each type of road in each grid)
M3^a^	Density of population and road network: $e_{i,g} = E_i \times \frac{{\frac{{r_g}}{{ \sum _{g = 1}^n {r_g} }} \times \frac{{p_g}}{{\mathop {\sum}_{g = 1}^n {p_g} }}}}{{ \sum \nolimits_{g = 1}^n {\left( {\frac{{r_g}}{{\sum _{g = 1}^n {r_g} }} \times \frac{{p_g}}{{\mathop {\sum}_{g = 1}^n {p_g} }}} \right)} }}$
M4-1^17^	Density of road network, multiplied by VKT allocation weights: Highways (52%), National roads (29%), Provincial roads (11%), Other roads (8%)
M4-2^16^	Density of road network, multiplied by VKT allocation weights: Highways (39%), National roads (25%), Provincial roads (19%), Other roads (17%)
M4-3^b^	Density of road network, multiplied by VKT allocation weights: Highways (58%), National roads (19%), Provincial roads (11%), Other roads (12%)
M4-4	Density of road network, multiplied by VKT allocation weights: Highways (52%), National roads (18%), Provincial roads (19%), Other roads (11%)

^a^ *e*_*i*,*g*_ is the 0.01˚ gridded HDT emissions for pollutant *i* and grid *g*. *E*_*i*_ is the total amount of HDT emissions for pollutant *i* in the BTH region. *p*_*g*_ is the number of populations in grid *g*. *r*_*g*_ is the length of the road in grid *g*.

^b^ The VKT allocation weights of M4-3 and M4-4 are the optimized results based on M4-1 and M4-2 by machine learning^32^.

### Reporting summary

Further information on research design is available in the Nature Research Reporting Summary linked to this article.

## Supplementary information

Reporting Summary

## Data Availability

The source data underlying Figs. 1a–b, 1a, 3a, 4a–f, 5a–d, 6e and 8 are provided as a source data file.xlsx. The Opmode emission rates, test HDT information in model validation, GDP and freight volume in Beijing, and the number of new HDTs in the BTH region are also provided in the source data file.xlsx. The source data file.xlsx is available online through the permanent repository under an Apache license 2.0 (https://github.com/fanyuandeng/TrackATruck). The GDP, freight volume and new HDT registration in Beijing and BTH region are also available online through NBSC website (http://www.stats.gov.cn/english). The population data used in this study is available on the LandScan website (https://landscan.ornl.gov/landscan-datasets). The road network used in this study is provided by the National Platform for Common Geospatial Information Services (https://www.tianditu.gov.cn), which is available after applying and obtaining permission.

## Source data

Source Data

## Code availability

The code of TrackATruck model validation and corresponding test data are available online through the permanent repository under an Apache license 2.0 (https://github.com/fanyuandeng/TrackATruck). The R package of optimized method by Ye^32^ is available on CRAN of R project (https://cran.r-project.org/web/packages/Rsolnp/index.html)^48^. Other R packages are also mentioned in the permanent repository.

## References

[1] Edenhofer, O. *Climate Change 2014: Mitigation of Climate Change* Vol. 3 (Cambridge University Press, 2015).

[2] Olivier J, Bouwman A, Van der Hoek K, Berdowski J (1998). Global air emission inventories for anthropogenic sources of NO_*x*_, NH_3_ and N_2_O in 1990. Environ. Pollut..

[3] Song C (2017). Air pollution in China: status and spatiotemporal variations. Environ. Pollut..

[4] Gao J (2018). Temporal-spatial characteristics and source apportionment of PM_2.5_ as well as its associated chemical species in the Beijing–Tianjin–Hebei region of China. Environ. Pollut..

[5] Ministry of Ecology and Environment of the People’s Republic of China. China *Vehicle Environmental Management Annual Report* (Ministry of Ecology and Environment of the People’s Republic of China, 2018).

[6] Liu H (2016). Health and climate impacts of ocean-going vessels in East Asia. Nat. Clim. Change.

[7] Liu H (2018). The impact of marine shipping and its DECA control on air quality in the Pearl River Delta, China. Sci. Total Environ..

[8] Zhang Q (2020). A WRF-Chem model-based future vehicle emission control policy simulation and assessment for the Beijing–Tianjin–Hebei region, China. J. Environ. Manag..

[9] Schnell JL (2019). Air quality impacts from the electrification of light-duty passenger vehicles in the United States. Atmos. Environ..

[10] Hata H, Tonokura K (2019). Impact of next-generation vehicles on tropospheric ozone estimated by chemical transport model in the Kanto region of Japan. Sci. Rep..

[11] Zheng B (2017). Resolution dependence of uncertainties in gridded emission inventories: a case study in Hebei, China. Atmos. Chem. Phys..

[12] Frey, H. C., Bharvirkar, R. & Zheng, J. Quantitative analysis of variability and uncertainty in emissions estimation. *Prepared by North Carolina State University for the US Environmental Protection Agency* (Research Triangle Park, NC, 1999).

[13] Miller, J. & Jin, L. *Global Progress Toward Soot-free Diesel Vehicles in 2018* (ICCT, Washington, DC, USA, 2018).

[14] Huo H, Wang M (2012). Modeling future vehicle sales and stock in China. Energy Policy.

[15] Huo H (2011). Modeling vehicle emissions in different types of Chinese cities: Importance of vehicle fleet and local features. Environ. Pollut..

[16] Yang, X., Liu, H., Man, H., & He, K. Characterization of road freight transportation and its impact on the national emission inventory in China. *Atmos. Chem. Phys.* **15**, 2105–2118 (2015).

[17] Zheng, B. et al. High-resolution mapping of vehicle emissions in China in 2008. *Atmos. Chem. Phys.* **14**, 9787–9805 (2014).

[18] Jalkanen J-P (2009). A modelling system for the exhaust emissions of marine traffic and its application in the Baltic Sea area. Atmos. Chem. Phys..

[19] Johansson L, Jalkanen J-P, Kukkonen J (2017). Global assessment of shipping emissions in 2015 on a high spatial and temporal resolution. Atmos. Environ..

[20] Li Meng, Zhang Qiang, Kurokawa Jun-ichi, Woo Jung-Hun, He Kebin, Lu Zifeng, Ohara Toshimasa, Song Yu, Streets David G., Carmichael Gregory R., Cheng Yafang, Hong Chaopeng, Huo Hong, Jiang Xujia, Kang Sicong, Liu Fei, Su Hang, Zheng Bo (2017). MIX: a mosaic Asian anthropogenic emission inventory under the international collaboration framework of the MICS-Asia and HTAP. Atmospheric Chemistry and Physics.

[21] Kurokawa J (2013). Emissions of air pollutants and greenhouse gases over Asian regions during 2000–2008: Regional Emission inventory in ASia (REAS) version 2. Atmos. Chem. Phys..

[22] Holman C, Harrison R, Querol X (2015). Review of the efficacy of low emission zones to improve urban air quality in European cities. Atmos. Environ..

[23] Wu Y (2017). On-road vehicle emissions and their control in China: a review and outlook. Sci. Total Environ..

[24] Li X (2015). Accuracy and reliability of multi-GNSS real-time precise positioning: GPS, GLONASS, BeiDou, and Galileo. J. Geod..

[25] Song L (2017). PM_2.5_ emissions from different types of heavy-duty truck: a case study and meta-analysis of the Beijing–Tianjin–Hebei region. Environ. Sci. Pollut. Res..

[26] Yang W (2018). High-resolution vehicle emission inventory and emission control policy scenario analysis, a case in the Beijing–Tianjin–Hebei (BTH) region, China. J. Clean. Prod..

[27] Jing B (2016). Development of a vehicle emission inventory with high temporal–spatial resolution based on NRT traffic data and its impact on air pollution in Beijing–Part 1: development and evaluation of vehicle emission inventory. Atmos. Chem. Phys..

[28] Yang D (2019). High-resolution mapping of vehicle emissions of atmospheric pollutants based on large-scale, real-world traffic datasets. Atmos. Chem. Phys..

[29] Cheng S, Lu F, Peng P (2020). A high-resolution emissions inventory and its spatiotemporal pattern variations for heavy-duty diesel trucks in Beijing, China. J. Clean. Prod..

[30] Zhang B, Wu S, Cheng S, Lu F, Peng P (2019). Spatial characteristics and factor analysis of pollution emission from heavy-duty diesel trucks in the Beijing–Tianjin–Hebei region, China. Int. J. Environ. Res. Public Health.

[31] Gong M (2017). Refined 2013-based vehicle emission inventory and its spatial and temporal characteristics in Zhengzhou, China. Sci. Total Environ..

[32] Ye, Y. Interior Algorithms for Linear, Quadratic, and Linearly Constrained Non-linear Programming. Ph.D thesis, Department of ESS, Stanford University (1987).

[33] Gately CK, Hutyra LR, Peterson S, Wing IS (2017). Urban emissions hotspots: quantifying vehicle congestion and air pollution using mobile phone GPS data. Environ. Pollut..

[34] Olivier, J. G. et al. Description of EDGAR Version 2.0: A set of global emission inventories of greenhouse gases and ozone-depleting substances for all anthropogenic and most natural sources on a per country basis and on 1 degree x 1 degree grid. (1996).

[35] Bahreini R., Middlebrook A. M., de Gouw J. A., Warneke C., Trainer M., Brock C. A., Stark H., Brown S. S., Dube W. P., Gilman J. B., Hall K., Holloway J. S., Kuster W. C., Perring A. E., Prevot A. S. H., Schwarz J. P., Spackman J. R., Szidat S., Wagner N. L., Weber R. J., Zotter P., Parrish D. D. (2012). Gasoline emissions dominate over diesel in formation of secondary organic aerosol mass. Geophysical Research Letters.

[36] Anenberg SC (2017). Impacts and mitigation of excess diesel-related NO_*x*_ emissions in 11 major vehicle markets. Nature.

[37] Sun Y (2013). Aerosol composition, sources and processes during wintertime in Beijing, China. Atmos. Chem. Phys..

[38] Wehner B (2008). Relationships between submicrometer particulate air pollution and air mass history in Beijing, China, 2004–2006. Atmos. Chem. Phys..

[39] Zhao P (2013). Characteristics of concentrations and chemical compositions for PM_2.5_ in the region of Beijing, Tianjin, and Hebei, China. Atmos. Chem. Phys..

[40] Koupal, J., Beardsley, M., Brzezinski, D., Warila, J. & Faler, W. US EPA’s MOVES2010 vehicle emission model: overview and considerations for international application. *Ann Arbor, MI: US Environmental Protection Agency, Office of Transportation and Air Quality*. http://www.epa.gov/oms/models/moves/MOVES2010a/paper137-tap2010.pdf (2010).

[41] Jimenez-Palacios, J. L. *Understanding And Quantifying Motor Vehicle Emissions With Vehicle Specific Power And Tildas Remote Sensing* (Massachusetts Institute of Technology, 1998).

[42] Davis N, Lents J, Osses M, Nikkila N, Barth M (2005). Development and application of an international vehicle emissions model. Transportation Res. Rec..

[43] Liu H (2007). Comparison of vehicle activity and emission inventory between Beijing and Shanghai. J. Air Waste Manag. Assoc..

[44] Liu H, He K, Barth M (2011). Traffic and emission simulation in China based on statistical methodology. Atmos. Environ..

[45] Liu H, He K, Lents JM, Wang Q, Tolvett S (2009). Characteristics of diesel truck emission in China based on portable emissions measurement systems. Environ. Sci. Technol..

[46] Liu H, Barth M (2012). Identifying the effect of vehicle operating history on vehicle running emissions. Atmos. Environ..

[47] Song C (2018). Heavy-duty diesel vehicles dominate vehicle emissions in a tunnel study in northern China. Sci. Total Environ..

[48] Ghalanos, A. & Theussl, S. *Rsolnp: General Non-linear Optimization Using Augmented Lagrange Multiplier Method* R package version 1 (Scientific Research, 2012).

