# Supplementary material for: A big data approach to improving the vehicle emission inventory in China
Source: Nat Commun. 2020 Jun 3;11:2801. doi: 10.1038/s41467-020-16579-w (PMC7271216; doi:10.1038/s41467-020-16579-w)
Supplement: Supplementary file 1 — Reporting Summary [file 41467_2020_16579_MOESM1_ESM.pdf]

natureresearch

Corresponding author(s): Huan Liu

Last updated by author(s): May 8, 2020

Reporting Summary

Nature Research wishes to improve the reproducibility of the work that we publish. This form provides structure for consistency and transparency in reporting. For further information on Nature Research policies, see Authors & Referees and the Editorial Policy Checklist.

Statistics

For all statistical analyses, confirm that the following items are present in the figure legend, table legend, main text, or Methods section.

n/a	Confirmed	
☐	☒	The exact sample size (n) for each experimental group/condition, given as a discrete number and unit of measurement
☒	☐	A statement on whether measurements were taken from distinct samples or whether the same sample was measured repeatedly
☒	☐	The statistical test(s) used AND whether they are one- or two-sided<br>*Only common tests should be described solely by name; describe more complex techniques in the Methods section.*
☒	☐	A description of all covariates tested
☒	☐	A description of any assumptions or corrections, such as tests of normality and adjustment for multiple comparisons
☐	☒	A full description of the statistical parameters including central tendency (e.g. means) or other basic estimates (e.g. regression coefficient) AND variation (e.g. standard deviation) or associated estimates of uncertainty (e.g. confidence intervals)
☒	☐	For null hypothesis testing, the test statistic (e.g. F, t, r) with confidence intervals, effect sizes, degrees of freedom and P value noted<br>*Give P values as exact values whenever suitable.*
☒	☐	For Bayesian analysis, information on the choice of priors and Markov chain Monte Carlo settings
☒	☐	For hierarchical and complex designs, identification of the appropriate level for tests and full reporting of outcomes
☐	☒	Estimates of effect sizes (e.g. Cohen's d, Pearson's r), indicating how they were calculated

Our web collection on statistics for biologists contains articles on many of the points above.

Software and code

Policy information about availability of computer code

Data collection	No software
Data analysis	R version 3.5.1

For manuscripts utilizing custom algorithms or software that are central to the research but not yet described in published literature, software must be made available to editors/reviewers. We strongly encourage code deposition in a community repository (e.g. GitHub). See the Nature Research guidelines for submitting code & software for further information.

Data

Policy information about availability of data

All manuscripts must include a data availability statement. This statement should provide the following information, where applicable:

- Accession codes, unique identifiers, or web links for publicly available datasets
- A list of figures that have associated raw data
- A description of any restrictions on data availability

The source data underlying Figs 1a-b, 2a, 3a, 4a-f, 5a-d, 6e, and 8 are provided as a source data file.xlsx. The Opmode emission rates, test HDT information in model validation, GDP and freight volume in Beijing, and the number of new HDTs in the BTH region are also provided in the source data file.xlsx. The source data file.xlsx is available online through the permanent repository under an Apache license 2.0 (https://github.com/fanyuandeng/TrackATruck). The GDP, freight volume, and new HDT registration in Beijing and BTH region are also available online through NBSC website (http://www.stats.gov.cn/english). The population data used in this study is available on the LandScan website (https://landscan.ornl.gov/landscan-datasets). The road network used in this study is provided by the National Platform for Common Geospatial Information Services (https://www.tianditu.gov.cn), which is available after applying and obtaining permission.

Field-specific reporting

Please select the one below that is the best fit for your research. If you are not sure, read the appropriate sections before making your selection.

☐ Life sciences ☐ Behavioural & social sciences ☒ Ecological, evolutionary & environmental sciences

For a reference copy of the document with all sections, see nature.com/documents/nr-reporting-summary-flat.pdf

Ecological, evolutionary & environmental sciences study design

All studies must disclose on these points even when the disclosure is negative.

Study description	This study proposes a new method and applies it to calculate the emission inventory of heavy-duty trucks (HDTs) in Beijing-Tianjin-Hebei (BTH) region in North China. Truck's trajectory data were used to calculated the air pollutant emissions. All truck emissions are aggregated according to vehicle information (e.g. emission standard). This study discusses these statistical results and the significance of the proposed method.
Research sample	The HDT travel data by BeiDou Navigation Satellite System (BDS) were provided by the Nationwide Road Freight Vehicles Public Supervision and Service platform of SINOIOV (SINOIOV platform). The details of HDT travel data include the recorded time, latitude, longitude, and truck's basic information, e.g., model year. The population data, road network, GDP, freight volumes, new HDT registration in BTH region were also used in this study. The population data were provided by LandScan database. The original population data is a raster map with population density. The road network data were provided by the National Platform for Common Geospatial Information Services. The original road network data includes the several road link information, e.g., the road types. The GDP, freight volume, and new HDT registration were provided by the National Bureau of Statistics of China (NBSC), which are the simple statistical values.
Sampling strategy	The whole fleet driving in the BTH region.
Data collection	All BDS data were sent instantly by BDS terminal equipment set up on the HDTs. The SINOIOV platform collected and managed these data. F.D. collected the BDS data by the off-line storage disk.
Timing and spatial scale	The timing scale is 1st Jan. in 2017 to 31st Dec. in 2018. The spatial scale is the Beijing-Tianjin-Hebei (BTH) region of China. In the timing-spatial scale, the BDS terminals set on all HDTs frequently sent the BDS signals to the SINOIOV platform. The transmission frequencies of the most terminals (>= 80 %) are higher than 1/30 Hz. When this study was conducted, the available BDS data was only updated to the end of 2018, so we set the stop time of sampling at that time.
Data exclusions	Only if HDT emission standards are not known, this data will be regarded as abnormal data and excluded. QA/QC show that is a very small probability event (<0.01%).
Reproducibility	This study did not involve experiments.
Randomization	HDT emissions are grouped according to emission standard, registered place and HDT activity location. Because the HDT emissions by these variables are significant, we do not control these variables.
Blinding	Since the analysis between the experimental group and the control group is not involved, this study was not relevant with extent of blinding.

Did the study involve field work? ☐ Yes ☒ No

Reporting for specific materials, systems and methods

We require information from authors about some types of materials, experimental systems and methods used in many studies. Here, indicate whether each material, system or method listed is relevant to your study. If you are not sure if a list item applies to your research, read the appropriate section before selecting a response.

Materials & experimental systems

n/a	Involved in the study	
☒	☐	Antibodies
☒	☐	Eukaryotic cell lines
☒	☐	Palaeontology
☒	☐	Animals and other organisms
☒	☐	Human research participants
☒	☐	Clinical data

Methods

n/a	Involved in the study	
☒	☐	ChIP-seq
☒	☐	Flow cytometry
☒	☐	MRI-based neuroimaging
